# Development and *in vivo* Assessment of a Rapidly Collapsible Anastomotic Guide for Use in Anastomosis of the Small Intestine: A Pilot Study Using a Swine Model

**DOI:** 10.3389/fsurg.2020.587951

**Published:** 2020-11-11

**Authors:** Alisha P. Pedersen, Karrer M. Alghazali, Rabab N. Hamzah, Pierre-Yves Mulon, Megan McCracken, Rebecca E. Rifkin, Anwer Mhannawee, Zeid A. Nima, Christopher Griffin, Robert L. Donnell, Alexandru S. Biris, David E. Anderson

**Affiliations:** ^1^Department of Large Animal Clinical Sciences, College of Veterinary Medicine, University of Tennessee, Knoxville, TN, United States; ^2^Center for Integrative Nanotechnology Sciences, University of Arkansas at Little Rock, Little Rock, AR, United States; ^3^NuShores BioSciences LLC, Little Rock, AR, United States; ^4^Equine Hospital, Veterinary Health Center, University of Missouri College of Veterinary Medicine, Columbia, MO, United States; ^5^Department of Biomedical and Diagnostic Sciences, College of Veterinary Medicine, University of Tennessee, Knoxville, TN, United States

**Keywords:** end-to-end anastomosis, anastomotic guide, side-to-side anastomosis, polyvinylpyrrolidone, polyurethane

## Abstract

Various conditions in human and veterinary medicine require intestinal resection and anastomosis, and complications from these procedures are frequent. A rapidly collapsible anastomotic guide was developed for small intestinal end-to-end anastomosis and was investigated in order to assess its utility to improve the anastomotic process and to potentially reduce complication rates. A complex manufacturing method for building a polymeric device was established utilizing biocompatible and biodegradable polyvinylpyrrolidone and polyurethane. This combination of polymers would result in rapid collapse of the material. The guide was designed as a hollow cylinder composed of overlaying shingles that separate following exposure to moisture. An *in vivo* study was performed using commercial pigs, with each pig receiving one standard handsewn anastomosis and one guide-facilitated anastomosis. Pigs were sacrificed after 13 days, at which time burst pressure, maximum luminal diameter, and presence of adhesions were assessed. Burst pressures were not statistically different between treatment groups, but *in vivo* anastomoses performed with the guide withstood 10% greater luminal burst pressure and maintained 17% larger luminal diameter than those performed using the standard handsewn technique alone. Surgeons commented that the addition of a guide eased the performance of the anastomosis. Hence, a rapidly collapsible anastomotic guide may be beneficial to the performance of intestinal anastomosis.

## Introduction

Small intestinal anastomosis is a relatively common procedure that may be performed in either emergency or elective situations and commonly involves resection of a diseased or damaged segment of the bowel (1–5). Numerous pathological conditions indicate the need for an intestinal anastomosis, including vascular compromise, bowel gangrene, obstruction, intussusception, volvulus, polyps, neoplasia, impaction, perforation due to trauma, severe inflammatory bowel disease refractory to medical therapy, chronic constipation, various congenital abnormalities, and severe inflammation due to disease. There are several techniques for performing an intestinal anastomosis. The operative technique chosen is at the discretion of the surgeon and is often based on the particular situation, personal preference, benefits or hindrances of specific techniques, cost, feasibility, availability of instruments, the diameter of the affected area of bowel, presence or lack of edema, location within the abdominal cavity, type of disease or condition, and time constraints (1, 2). Regardless of the techniques used, practices that provide the best post-operative recovery include adequate accessibility of the affected bowel segment, gentle manipulation of the bowel and surrounding abdominal structures, appropriate hemostasis and maintenance of vascularization following transection, avoidance of tension at the anastomotic site, proper surgical technique, and prevention of contamination of the abdomen with intestinal contents (2).

The most common anastomotic techniques can be divided into two broad categories, handsewn and stapled, within which are numerous sub-categories. Categories of handsewn anastomoses include simple continuous suture pattern vs. interrupted suture pattern (5–8); single-layered or double-layered closure (9–11); inverting, everting, or appositional pattern (12–15); end-to-end (EEA) or side-to-side (SSA) positioning of intestinal segments; use of absorbable vs. non-absorbable suture material and choice of a specific type of suture material; extramucosal or full-thickness suturing; and choice of spacing between suture placements (16, 17). Categories of stapled anastomoses include: end-to-end or side-to-side positioning; oversewing the stapled area or burying it; and choice of stapling device used (1). No matter the technique, several potential complications may occur during or after an intestinal anastomosis procedure, some of which are life-threatening. A complication that may present itself early in the recovery period is leakage from the anastomotic site. During the first 5–7 days of recovery, the efficacy of the anastomotic site largely relies on the integrity of the suture material or staples to holdfast in the tissues. Leakage that occurs within the first day or two after surgery is often associated with the techniques utilized to perform the anastomosis. If leakage occurs beyond the first 5–7 days in the postoperative recovery period, it is more likely to be associated with poor intestinal healing (2). Leakage may take the form of diffuse peritonitis or localized abscess formation. Peritonitis has a high morbidity and mortality rate and requires additional surgical intervention (2). Leakage has been reported to increase the expected mortality rate after bowel anastomosis from 7.2 to 22% (1, 18).

Another commonly encountered complication is excessive bleeding from the anastomotic site, either intraoperatively or postoperatively. The integrity of the anastomosis should be re-evaluated if this occurs and hemostasis achieved as needed. Postoperative bleeding can be evident as hematemesis, melena, bleeding through an intra-abdominal drain, progressive anemia, and abdominal distension, among other signs. These cases may need to be treated with medical management or, if persistent or severe, surgical intervention. Stapled anastomoses in particular have been shown to result in disruption of mesenteric blood vessels, increasing the risk of ischemia of the bowel (2). Stricture of the intestine at the anastomosis is a serious complication that has been reported to occur more frequently after stapled EEA than handsewn EEA (2, 19). Medical management of anastomotic leakage after surgery is a significant risk factor contributing to the development of a stricture, and dilatation or surgical revision may be necessary to treat this complication (2).

We hypothesized that the use of an anastomotic guide (AG), placed within the lumen of the intestine during surgery would improve the accuracy of EEA by providing a means to appose the cut ends of the intestine so that precise sutures could be placed. This precision surgery would result in increased lumen diameter and reduced potential for leakage after anastomosis. The device was designed such that it would rapidly collapse after surgery so as not to be predisposed to complications associated with other intraluminal intestinal devices. Intraluminal stents have been used to expand and maintain the lumen size of strictured bowel after colon resection and anastomosis. To date, intestinal stents used to expand the intestinal wall contain non-degradable or slowly degraded materials (20). Intestinal stents may increase morbidity rates associated with interruption of intestinal motility, impaction of the stent by digesta, stent migration, and re-obstruction (20–22). A slowly degrading (up to 3 months) intraluminal colonic stent was described for treatment of strictures of the colon after anastomotic leakage (23). A rapidly degraded or collapsed intraluminal device would eliminate post-operative morbidity associated with the use of the device. We aimed to assess the feasibility of a rapidly collapsible, intraluminal small intestinal AG to reduce the potential for post-operative complications, as well as to improve the accuracy and efficiency of the anastomotic procedure (23). A prototype AG was fabricated and underwent numerous characterization assessments prior to application in an *in vivo* swine model, which was established in order to assess post-surgical complications when compared with a standard handsewn EEA method.

## Materials and Methods

### Anastomotic Guide Composition and Fabrication

Non-degradable 3D-printed models of an intraluminal guide were initially fabricated based on expected bowel size in an ~70 kg pig, as well as the length predicted to be of greatest benefit to the technical performance of an anastomosis. A hollow cylindrical tube was determined to be the ideal shape. These prototypes were used as models for creation of a rapidly collapsible, intraluminal AG. The desired specifications were that the guide would collapse no <30 min and no longer than 3 h after implantation within the intestine.

**Figure 1:**
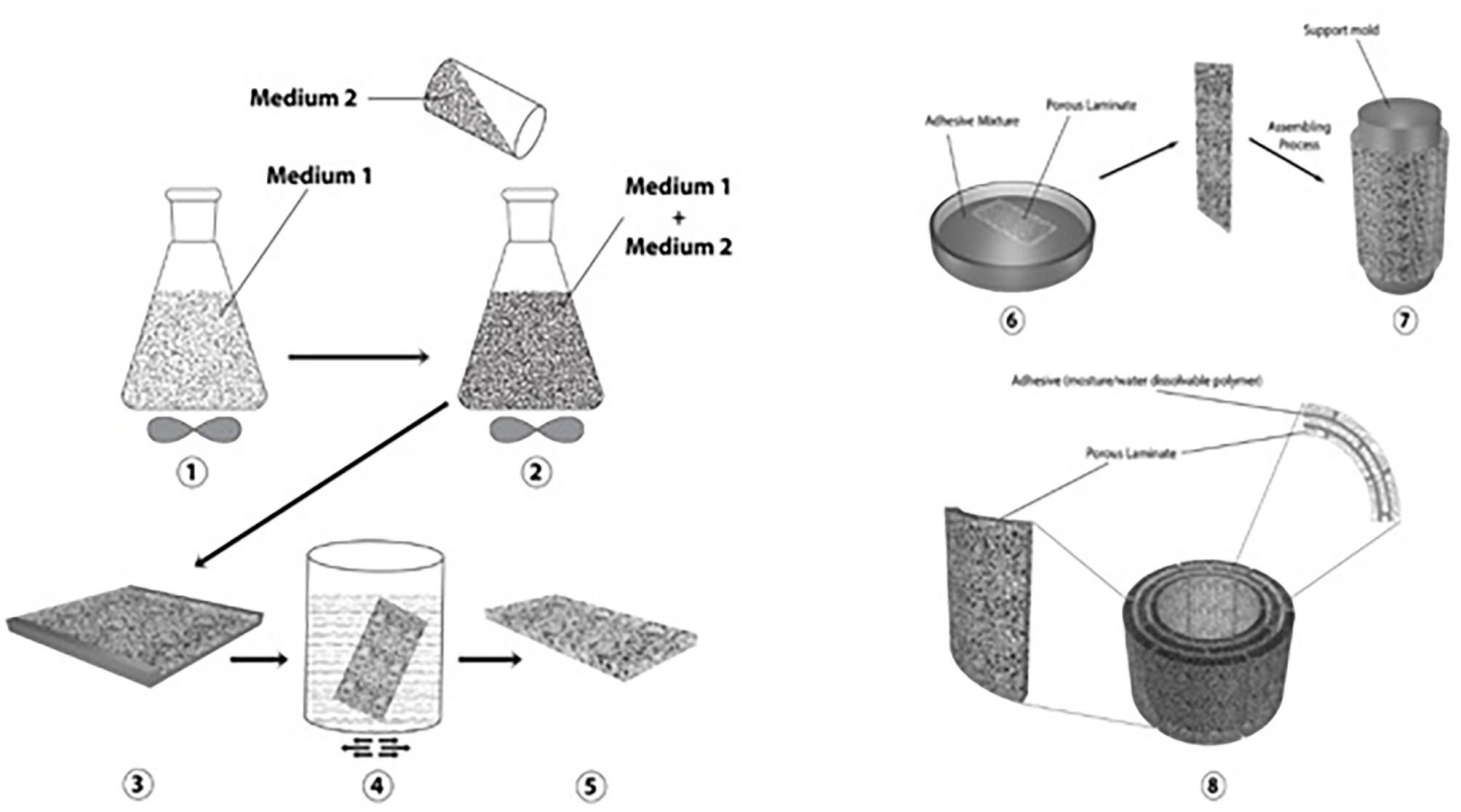
General protocol used to fabricate the device. Medium 1 refers to PU dissolved in 90/10 ethanol/deionized water. Medium 2 refers to salt porosity agents with 75–150 pm diameter.

**Figure 2:**
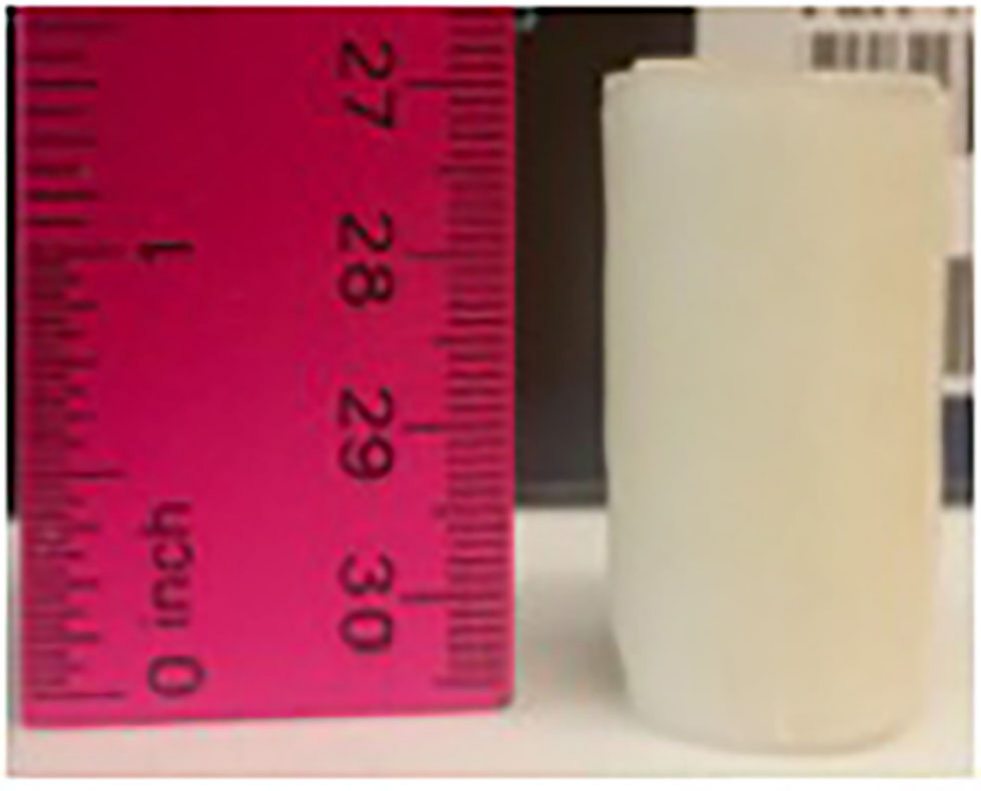
Fabricated device measuring 3 × 1.5 cm.

A guide (patent pending: PCT/US2019/041550) was fabricated using a hollow cylindrical tube composed of layers of biocompatible polymer polyurethane (PU) (HydroMed:AdvanSource Biomaterials; Wilmington, MA) and moisture/fluid-degradable polymer polyvinylpyrrolidone (PVP) (polyvinylpyrrolidone: Sigma-Aldrich: Average MW 10000, St. Louis, MO). These polymers were chosen based on their water responses (water uptake and ability to dissolve in water). The polymer layers were produced using a modified salt leaching method. Briefly, the PU polymer was dissolved in 90/10 ethanol/deionized water to form medium 1, then 10 g of 75–150-μm particles (porosity agents — medium 2) for each 1 g of PU were added (Figure 1). The material was mixed extensively, poured over a glass mold, and transferred into a water bath to remove the salt particles. The resulting polymer film was dried and cut into a small laminate (3 × 1.5 cm). Next, the porous polymer laminate was saturated with PVP. The polymer laminates were then assembled to form multilayers over the support mold. The mold was removed, and the samples were left to dry (Figure 2).

The device was fabricated to serve as a temporary supportive intraluminal anastomotic guide that can rapidly lose its integrity after becoming wet and within the desired time. The desired specifications were that the guide would lose its integrity in no <30 min and no more than 3 h after implantation within the intestine. To test the device's ability to meet these specifications, the fabricated samples were immersed in a water bath, and the integrality was observed over time.

### *In vivo* Investigation

**Figure 3:**
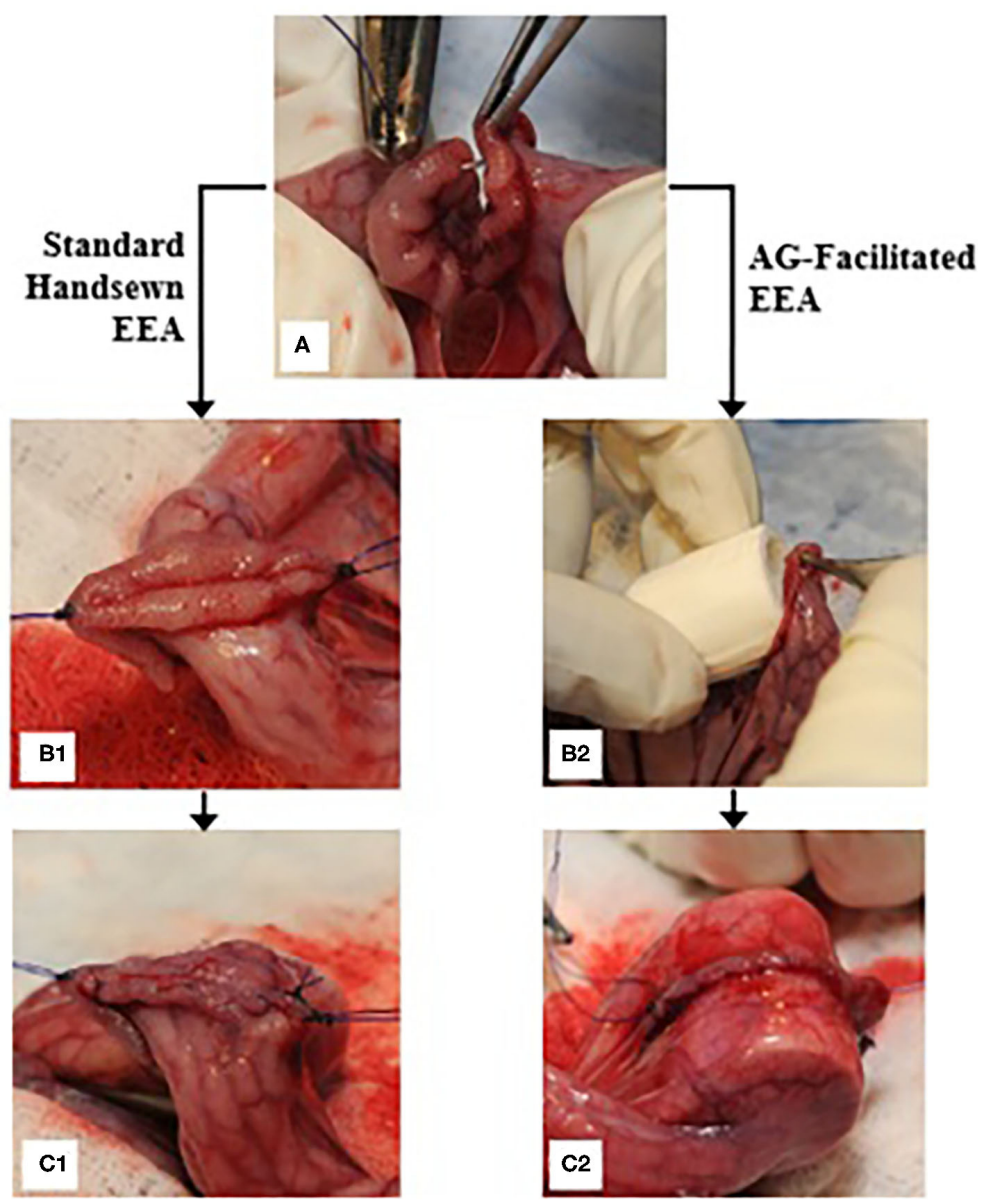
End-to-end anastomosis procedure; **(A)** a single interrupted suture is placed on the anti-mesenteric margin of the bowel immediately following performance of a transverse enterotomy; **(B1)** single simple interrupted sutures are secured on both the anti-mesenteric and mesenteric margins, and the bowel edges are apposed for further suturing; **(C1)** a row of simple continuous sutures is placed hemi-circumferentially; **(B2)** an AG is placed into the lumen of the bowel; **(C2)** the anastomosis is performed overtop the AG after complete placement within the lumen.

*In vivo* studies were done after approval by the Institutional Animal Care and Use Committee (IACUC protocol #2522) at the University of Tennessee, Knoxville. Six domestic cross-bred pigs, weighing 35 to 70 kg, were housed in separate adjacent pens and acclimated to their environment for 12 days. Each pig was fasted for a minimum of 12 h prior to surgery, and water access was restricted a minimum of 2 h before surgery. Peri-operative analgesia was provided by placement of transdermal fentanyl patches (1 μg/kg) along the dorsal midline in the mid-thoracic region at least 12 h prior to surgery. Subjects were pre-medicated with xylazine (2 mg/kg, IM), induced with a combination of midazolam (0.1–0.2 mg/kg, IM) and ketamine (10 mg/kg, IM), an endotracheal tube was placed, and anesthesia maintained using isoflurane (range 1 to 5%) vaporized into oxygen (100%). Each subject was placed into dorsal recumbency, clipped, and aseptically prepared along the ventral midline. The surgical model, briefly depicted in Figure 3, consisted of a 10 cm ventral midline laparotomy with subsequent exteriorization of 20–40 cm of jejunum. The bowel was milked free of intraluminal contents and a 15 cm segment was isolated with Doyen intestinal clamps. A complete, transverse enterotomy was performed at a 90° angle and single interrupted sutures of #3-0 PDS (Ethicon, INC. Somerville, New Jersey) were placed at the mesenteric and anti-mesenteric margins of the cut edges for stabilization and to aid in apposition of the edges. The anastomosis was completed with an interrupted simple continuous appositional pattern (two suture segments, each placed hemi-circumferentially) using #3-0 PDS. Integrity, blood perfusion, and complete closure of the anastomosis was evaluated. Approximately 20 cm distal to the first anastomosis, a second enterotomy was performed in like manner, except after the first single interrupted suture was placed and before closing the cut edges of the bowel with the same technique, the collapsible intraluminal anastomotic guide was placed within the lumen traversing and centered on the cut edges. Following replacement of the jejunum within the abdominal cavity, the linea alba was closed using #0 PDS, the subcutaneous layer with #2-0 PDS, and finally the skin with #1 polypropylene, all utilizing a simple continuous pattern. Surgeons were consulted regarding their subjective opinion of the utility of the AG during surgery.

Pigs received intramuscular ceftiofur (Excede, Zoetis Services LLC, Parsippany, New Jersey; 5 mg/kg dose) prior to surgery. The pigs were monitored frequently for signs of pain, incision site abnormalities, vomiting, abdominal distention, diarrhea, or constipation. Peri-operative analgesia was managed using fentanyl patches (1 μg/kg, TD, 72 h) and meloxicam (0.4 mg/kg, PO, q24 h × 5 d). Pigs were monitored for activity, appetite, and clinical signs of pain through day 13 at which time the study was terminated.

All pigs were sacrificed 13 days after surgery, and necropsy examinations performed to assess the gross appearance of the bowel and anastomoses, as well as the surrounding abdominal cavity. Burst pressure withstood by the anastomotic sites was determined by instilling saline into the anastomotic region and observing for leakage. Fluid pressure was assessed using a digital pressure monitor (Surgivet® V6400 Invasive Blood Pressure Monitor, Smiths Medical PLC, Minneapolis, MN). The vicinity of the anastomotic site was occluded using surgical clamps, leaving an ~12 cm long segment centered on the anastomosis. A 16-gauge needle and IV line were used to instill saline solution into one side of this region, and a second 16-gauge needle was placed into the opposing side and attached to the pressure monitor. The lumen was gradually distended with saline while the anastomosis was observed for leaks. Once a leak occurred, the pressure reading was recorded and considered the maximum burst pressure withstood by the anastomotic site for that sample.

The external diameter of the bowel was also measured for the assessment of stricture of the anastomotic site. Diameter difference was calculated based on diameter measurements of the intestinal regions just proximally and distally adjacent to the anastomosis, as well as at the anastomotic site, utilizing calipers while saline remained infused in the segments following burst pressure measurement. Histologic evaluation included hematoxylin and eosin and trichrome stains to assess fibrosis and collagen deposition, presence and characterization of inflammation at the anastomotic sites and within the adjacent tissue, approximate width of anastomotic sites, serosal thickness, and any additional abnormalities.

## Results

### Anastomotic Guide Characteristics

#### 3D Keyence Laser Microscope Analysis

**Figure 4:**
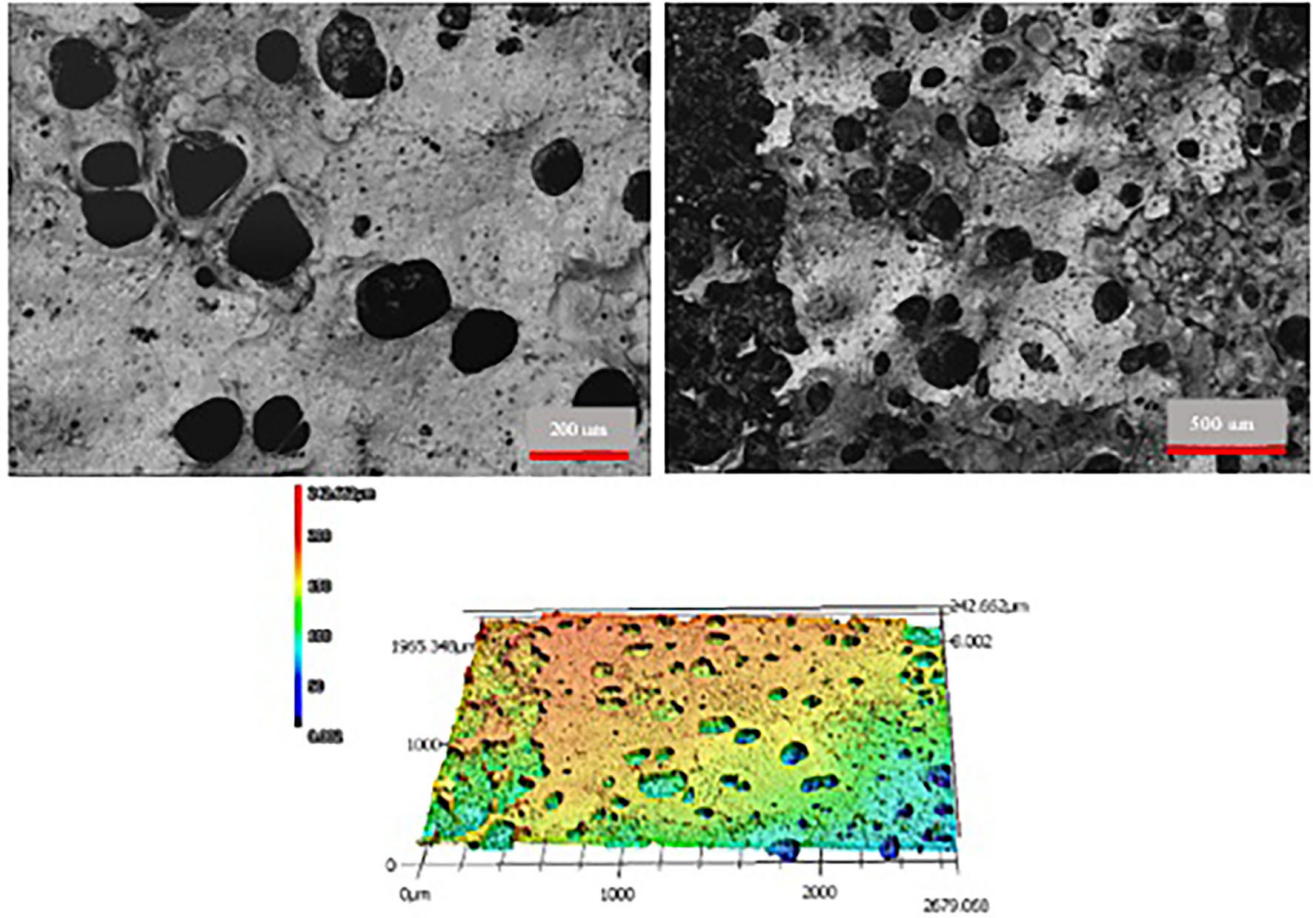
3D LSCM results for the porous polymer laminate used to fabricate the device.

Three-dimensional (3D) laser microscopy (LSCM, VK-X260K, Keyence, Itasca, IL) was used to evaluate the surface morphology and topography of the samples, allowing visualization of the porous structure of the polymer laminate. The porous polymer laminate was examined using 20X and 10X lenses. The data was analyzed with Keyence's Multi-File Analyzer software. 3D microscopy confirmed that the polymer laminate has a porous structure, as shown in Figure 4.

#### Device Testing

Generally, when dry, the device is a rigid structure due to the solidification of PVP. The fabricated samples were immersed in a water bath, and the integrality was observed over time. The device lost its integrity as a function of the water/fluid response of its two polymers, causing it to collapse.

### *In vivo* Investigation

Morbidities observed after surgery included minimal incidences of diarrhea, mild pyrexia that resolved after treatment with antibiotics, and mild swelling at the incision site. No remnants of the AGs were recovered in feces.

Following sacrifice of the pigs, gross examination of the anastomoses and surrounding abdominal cavity was performed. Adhesions were discovered at eleven out of the twelve EEA sites and at adjacent regions within the abdominal cavity in five out of the six pigs. There were no significant differences in adhesion development between the anastomotic sites that involved the AG and those that did not. A standard handsewn EEA in one pig was noted to have had minor dehiscence, and no leakage or dehiscence was noted in any of the EEA performed with the AG. The gross appearance of the healed margins of the bowel were similar for all EEA sites.

Burst pressure was found to be ~10% greater at AG-facilitated anastomotic sites than those of standard handsewn EEA sites (Table 1); however, this difference was not statistically significant. The maximum diameter achieved at the anastomotic sites that utilized an AG was significantly greater than that achieved with the standard handsewn anastomoses (Table 1). Subjective evaluation by surgeons performing the anastomoses noted that the guide aided in the placement of more evenly spaced sutures and eased the performance of the EEA. The surgeons noted that there was some difficulty placing the guide within the lumen due to its pliability (accountable to submersion in saline prior to surgery).

**Table 1:** Comparison of the average number of adhesions at the anastomotic site, burst pressure, and maximum diameter for each anastomotic technique.

	**Standard handsewn EEA**	**Anastomotic guide EEA**
Average number of adhesions at site	1	1
Average burst pressure (mmHg)^b^	150.6 ± 49.3	166.0 ± 47.5
Average maximum diameter at anastomotic site (mm)^a^	22.73 ± 2.0	26.59 ± 3.9
Diameter difference of anastomotic sites (%)^a^		+17%

*Burst pressure was obtained at only five of the six anastomotic sites of each technique due to perforation of the anastomotic site or adjacent bowel in two samples. Presence of adhesions at the anastomotic sites and local regions of the abdominal cavity was assessed grossly. Burst pressure was measured by instilling saline into the anastomotic region and observing the maximum pressure withstood by the anastomosis via a digital pressure monitor. Maximum diameter at each anastomotic site was measured while saline remained infused in the segments following burst pressure measurement. Diameter difference is the difference between the average diameter of the anastomoses performed with and without the use of an AG*.

a *Statistically significant difference (p < 0.05)*,

b *No significant difference*.

Histologic evaluation revealed characteristics of expected healing within all of the samples, including suture granulomas adjacent to anastomotic sites, fibrosis and collagen deposition within sites, serosal thickness at sites between 2 and 4 times that of the adjacent normal tissue, and sites ranging in width from <0.5 to 5 mm. All anastomotic sites contained a normal expected amount of mild-to-moderate inflammatory cell infiltration, typically mixed eosinophilic and lymphocytic inflammation. Two anastomotic sites (one standard and one AG) in two separate pigs appeared to have features of both jejunum and ileum, dependent on the section examined. The standard handsewn anastomosis in one pig demonstrated a focal region of ulceration and marked inflammatory infiltrates, including dead or degenerate segmented eosinophils and neutrophils. This sample demonstrated an increased presence of macrophages within an area of fibrosis. Within this same pig, the bowel edges of the AG site appeared to be overlapped in one region. Another standard handsewn anastomosis in a different pig similarly demonstrated an area of ulceration, along with the presence of hemorrhage, imbedded plant material or suture, and marked suppurative and eosinophilic inflammation. Hemorrhage was found within the serosa of this sample. Within the standard handsewn site of an additional pig, there was a focal region of pyogranulomatous inflammation, and in the AG site of this same pig, there was a mild-to-moderate amount of inflammation and hemorrhage within the serosa which was deemed likely not significant.

## Discussion

During post-mortem assessment, anastomotic site diameter was deemed to be improved in the sites in which an AG was used. Although small, this difference may be clinically significant, resulting in a decreased likelihood of stricture and impaction at surgical sites. A meta-analysis examining complications following sutured and stapled colorectal anastomosis in 1,233 human patients determined that strictures occurred in 2 and 8% of patients, respectively (1, 24). One limitation to evaluating the diameter difference by measuring the external diameter with calipers is that any inverted mucosa resulting in a further narrowed intraluminal diameter would not be accounted for. Two alternative methods of assessing the intraluminal diameter and anastomotic index are by instillation of a contrast agent into the delineated region of the anastomosis and subsequent radiographic imaging (25, 26), or by measurement of the wall thickness at the anastomotic site and proximally and distally to it utilizing calipers (26).

Differences in burst pressure between the groups were not significantly different. This suggests that the healing process in the intestine with EEA is similar regardless of the technique employed. Maximum burst pressures achieved were physiologically appropriate, and in fact were in excess to normal physiological pressures (27, 28), so it does not appear that the performance of anastomoses produced a risk of bowel disruption during motility, at least when assessed 2 weeks post-operatively.

Adhesion development occurred at nearly all anastomotic sites and within local areas of the abdominal cavity. It was difficult to differentiate which anastomotic site may have incited the additional adhesions within the abdomen, and the ~20 cm distance between the two anastomoses may ultimately have been too close in proximity to allow this determination. Intraluminal appearance of each anastomosis was not noticeably different, supporting the likelihood that the methods did not adversely affect the normal process of intestinal healing. One pig appeared to have developed a small dehiscence at the standard handsewn anastomotic site, which was sealed with an adhesion. Histologic evaluation of the samples did not reveal any substantial concerns in regards to integrity of the anastomotic sites or presence of excessive inflammation that would be expected to progress to significant disease, including within the samples that demonstrated focal regions of inflammation. All samples revealed anticipated indicators of healing, including granuloma formation at suture sites, fibrosis and collagen deposition within the anastomotic sites, and thickening of the serosa.

The EEA technique was noted by the surgeons to be easier to perform with the use of the AG. This is likely owing to the ability to place sutures more easily within the cut edges of bowel due to the edges being dilated by the guide as opposed to the natural contraction and eversion that occurs when the bowel is transected. Precision and accuracy in reconstruction of the continuity and patency of the bowel is critical to ensuring that devastating dehiscence or stricture does not occur (2, 29). The only concern noted with the use of the AG regarded difficulty placing the guide within the lumen due to its pliability. The guides were briefly soaked in saline prior to surgery, which likely accounts for the majority of this pliability. However, sturdiness of the guide may also be addressed in modified designs by altering the thickness or polymer composition. Time to collapse of the guide was assessed in hydration studies prior to placement within the subjects and was deemed appropriate. No remnants remained within the lumen upon necropsy evaluation, which further supports that the guides broke apart.

**Figure 5:**
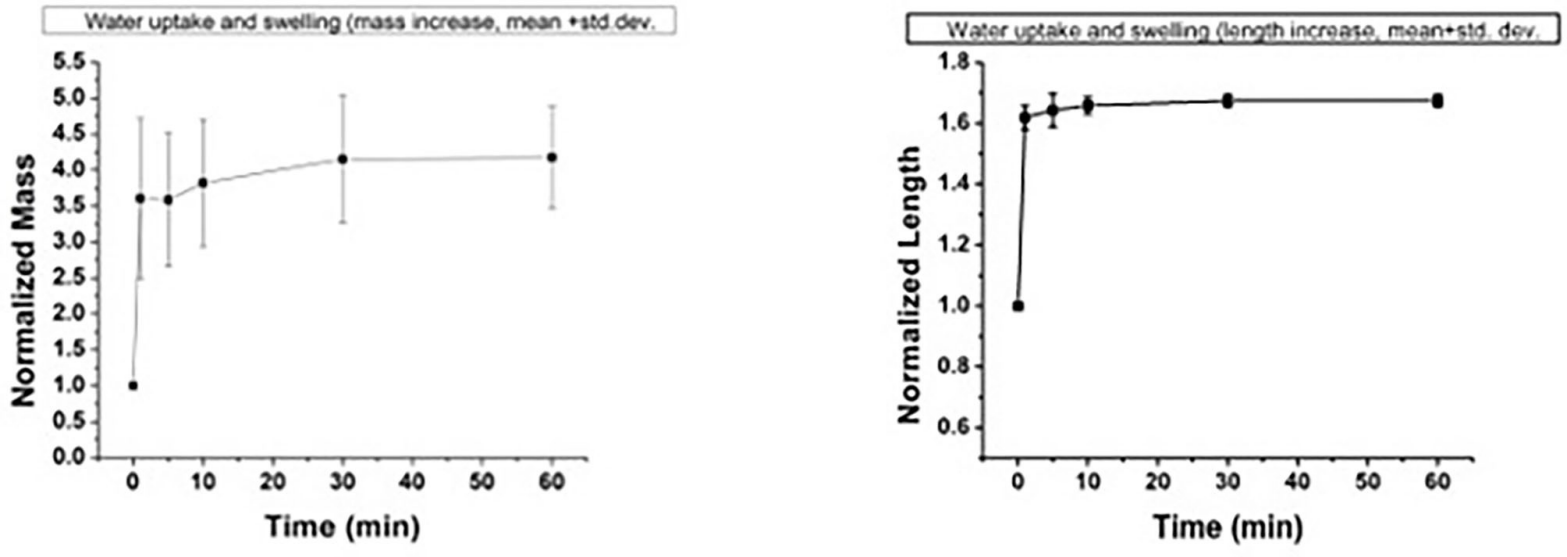
Water uptake and swelling behavior of the porous polymer laminate used to fabricate the device. **Left**: normalized mass; **right**: normalized length.

One concern about placement of a medical device within the bowel lumen is the potential for complications associated with the device itself. Non-degradable or slowly degrading intestinal stents that have been previously available or investigated may increase morbidity rates associated with hindrance to normal peristalsis, dislodgement, blockage of the intestinal lumen with the stent, and impaction of the lumen of the stent with digesta (20–22). We designed a rapidly collapsible polymeric device to avoid these potential complications. Should the guide dislodge shortly after the surgery, it would quickly collpase with the passage of digesta within the bowel. The testing of the device by water bath immersion demonstrated that the device lost its integrity over time as a function of the water/fluid response of its two polymers, causing it to collapse. Generally, the two polymers have different responses to fluid. PU uptakes fluid into its structure, with the ability to increase in mass by about 300% of its dry weight and expand in size by about 60%, as shown in Figure 5. In contrast, PVP dissolves when exposed to fluid, causing the device to lose its polymer-polymer bonds.

The ability of an intraluminal anastomotic guide to aid in increasing the diameter of an intestinal anastomosis site, as well as ease the performance of the technique itself, without presenting any additional complications, supports the use of guides for this particular procedure. This could ultimately reduce complications that occur post-operatively, including dehiscence, leakage, peritonitis, stricture, and impaction. Any reduction in time of performance would also be beneficial as some patients undergoing this procedure may be physiologically and anesthetically unstable. The use of a swine model is advantageous for translation to human medicine, as swine have gastrointestinal tracts that are comparable to humans. Continued research is warranted to develop a collapsible or degradable intraluminal guide for small intestinal anastomosis for use in human and animal patients, and the data from this study will be utilized in the planning of a follow-up validation study employing a larger number of swine with assignment of animals to a single treatment group rather than the performance of both procedures within the same animal.

## Data Availability Statement

The raw data supporting the conclusions of this article will be made available by the authors, without undue reservation.

## Ethics Statement

The animal study was reviewed and approved by Institutional Animal Care and Use Committee at the University of Tennessee, Knoxville.

## Author Contributions

AP contributed to the design of the device and performed all of the animal studies. KA and AB contributed to the design and performed all of the fabrication, characterization, and testing of the anastomotic guide. AP and KA wrote the manuscript. RH, AM, ZN, and CG contributed to the development and testing of the anastomotic guide. DA, P-YM, MM, and RR contributed to the application of the device in the live animal model. AB and DA were mentors during the development and progression of the entire study. All authors contributed to the article and approved the submitted version.

## Conflict of Interest

AB and DA disclose that they are co-founders of NuShores BioSciences LLC. Although KA is currently employed by NuShores BioSciences, he was working as a graduate student at ULAR when he developed the technology and throughout the completion of this research. As such, research was conducted in the absence of any commercial or financial relationships that could be construed as a potential conflict of interest. The remaining authors declare that the research was conducted in the absence of any commercial or financial relationships that could be construed as a potential conflict of interest.

## Abbreviations

EEA: End-to-end anastomosis
SSA: Side-to-side anastomosis
AG: Anastomotic guide
PVP: Polyvinylpyrrolidone
PU: Polyurethane.

## References

[1] Goulder F. Bowel anastomoses: the theory, the practice and the evidence base. World J Gastrointest Surg. (2012) 4:208–13. 10.4240/wjgs.v4.i9.208 23293735 PMC3536859

[2] Medscape Intestinal Anastomosis Technique. (2016). Available online at: http://emedicine.medscape.com/article/1892319-technique (accessed June 30, 2017).

[3] Tobias KM Intestinal resection and anastomosis Manual of Small Animal Soft Tissue Surgery. Ames, IA: Wiley-Blackwell (2010). p. 175–182.

[4] Ralphs SC Jessen CR Lipowitz AJ. Risk factors for leakage following intestinal anastomosis in dogs and cats: 115 cases (1991-2000). J Am Vet Med Assoc. (2003) 223:665–82. 10.2460/javma.2003.223.73 12839067

[5] Weisman DL Smeak DD Birchard SJ Zweigart SL. Comparison of a continuous suture pattern with a simple interrupted pattern for enteric closure in dogs and cats: 83 cases (1991-1997). J Am Vet Med Assoc. (1999) 214:1507–10. 10340077

[6] Burch JM Franciose RJ Moore EE Biffl WL Offner PJ. Single-layer continuous versus two-layer interrupted intestinal anastomosis: a prospective randomized trial. Ann Surg. (2000) 231:832–7. 10.1097/00000658-200006000-00007 10816626 PMC1421072

[7] Ellison GW Jokinen MP Park RD End-to-end approximating intestinal anastomosis in the dog: a comparative fluorescein dye, angiographic, and histopathologic evaluation. J Am Anim Hosp Assoc. (1982) 18:729–36.

[8] Allen DA Smeak DD Schertel ER Prevalence of small intestinal dehiscence and associated clinical factors: a retrospective study of 121 dogs. J An Anim Hosp Assoc. (1992) 28:70–6.

[9] Shikata S Yamagishi H Taji Y Shimada T Noguchi Y Single- versus two-layer intestinal anastomosis: a meta-analysis of randomized controlled trials. BMC Surg. (2006) 6:2 10.1186/1471-2482-6-2 16438733 PMC1373646

[10] Garude K Tandel C Rao S Shah N. Single layered intestinal anastomosis: a safe and economic technique. Indian J Surg. (2013) 75:290–3. 10.1007/s12262-012-0487-7 24426455 PMC3726811

[11] Sajid MS Siddiqui MR Baig MK. Single layer versus double layer suture anastomosis of the gastrointestinal tract. Cochrane Database Syst Rev. (2012) 1:CD005477. 10.1002/14651858.CD005477.pub4 22258964 PMC12107684

[12] Getzen LC Roe RD Holloway CK. Comparative study of intestinal anastomotic healing in inverted and everted closures. Surg Gynecol Obstet. (1966) 123:1219–27. 5953952

[13] Goligher JC Morris C McAdam WA De Dombal FT Johnston D. A controlled trial of inverting versus everting intestinal suture in clinical large-bowel surgery. Br J Surg. (1970) 57:817–22. 10.1002/bjs.1800571106 4920295

[14] Holt DE “Large intestines”. In: Slatter DH, editor. Textbook of Small Animal Surgery, 3rd ed. Philadelphia, PA: Saunders (2003). p. 665–82.

[15] Bellenger CR. Comparison of inverting and appositional methods for anastomosis of the small intestine in cats. Vet Rec. (1982) 110:265–8. 10.1136/vr.110.12.265 7080414

[16] Deveney KE Way LW. Effect of different absorbable sutures on healing of gastrointestinal anastomoses. Am J Surg. (1977) 133:86–94. 10.1016/0002-9610(77)90199-4 835784

[17] Milovancev M Weisman DL Palmisano MP. Foreign body attachment to polypropylene suture material extruded into the small intestine lumen after enteric closure in three dogs. J Am Vet Med Assoc. (1982) 225:265–8. 15626221 10.2460/javma.2004.225.1713

[18] Fielding LP Stewart-Brown S Blesovsky L Kearney G. Anastomotic integrity after operation for large-bowel cancer: a multicentre study. Br Med J. (1980) 281:411–14. 10.1136/bmj.281.6237.411 7427298 PMC1713296

[19] Leung TTW MacLean AR Buie WD Dixon E. Comparison of stapled versus handsewn loop ileostomy closure: a meta-analysis. J Gastrointest Surg. (2008) 12:939–44. 10.1007/s11605-007-0435-1 18071833

[20] Wang Z Li N Li R Li Y Ruan L Biodegradable intestinal stents: a review. Pro Nat Sci-Mater. (2014) 24:423–32. 10.1016/j.pnsc.2014.08.008

[21] Brightwell NL McFee AS Aust JB. Bowel Obstruction and the Long Tube Stent. Arch Surg. (1977) 112:505–11. 10.1001/archsurg.1977.01370040157024 849159

[22] Son SR Franco RA Bae SH Min YK Lee BT. Electrospun PLGA/gelatin fibrous tubes for the application of biodegradable intestinal stent in rat model. J Biomed Mater Res Part B. (2013) 101B:1095–105. 10.1002/jbm.b.32923 23564699

[23] Wang Y Cai X Jin R Liang Y Huang D Peng S. Experimental study of primary repair of colonic leakage with a degradable stent in a porcine model. J Gastrointest Surg. (2011) 15:1995–2000. 10.1007/s11605-011-1593-8 21706291

[24] Lustosa SA Matos D Atallah AN Castro AA Stapled versus handsewn methods for colorectal anastomosis surgery. Cochrane Database Syst Rev. (2001) 3:CD003144 10.1002/14651858.CD003144.pub2 11687041

[25] Gandini M. *In vitro* evaluation of a closed-bowel technique for one-layer hand-sewn inverting end-to-end jejunojejunostomy in the horse. Vet Surg. (2006) 35:683–8. 10.1111/j.1532-950X.2006.00209.x 17026556

[26] Zilling TL Jansson O Walther BS Ottosson A. Sutureless Small bowel anastomoses: experimental study in pigs. Eur J Surg. (1999) 165:61–8. 10.1080/110241599750007522 10069636

[27] Coolman BR Ehrhart N Pijanowski G Ehrhart EJ Coolman SL. Comparison of skin staples with sutures for anastomosis of the small intestine of dogs. Vet Surg. (2000) 29:293–302. 10.1053/jvet.2000.7539 10917278

[28] Ellison GW End-to-end anastomosis in the dog: a comparison of techniques.” Comp Cont Ed Pract Vet. (1981) 3:486–95.

[29] Shandall A Lowndes R Young HL. Colonic anastomotic healing and oxygen tension. Br J Surg. (1985) 72:606–9. 10.1002/bjs.1800720808 3896373

